# Long Noncoding RNAs Responding to Ethanol Stress in Yeast Seem Associated with Protein Synthesis and Membrane Integrity

**DOI:** 10.3390/genes16020170

**Published:** 2025-01-28

**Authors:** Amanda Piveta Schnepper, Agatha M. S. Kubo, Camila Moreira Pinto, Ramon Hernany Martins Gomes, Matheus Naia Fioretto, Luís Antonio Justulin, Aline M. M. Braz, Marjorie de Assis Golim, Rejane M. T. Grotto, Guilherme Targino Valente

**Affiliations:** 1 Department of Bioprocess and Biotechnology, School of Agriculture, Sao Paulo State University (UNESP), Botucatu 18610-034, SP, Brazil; 2 Laboratory of Applied Biotechnology, School of Medicine, Sao Paulo State University (UNESP), Botucatu 18618-687, SP, Brazil; 3 Department of Structural and Functional Biology, Institute of Biosciences, Sao Paulo State University (UNESP), Botucatu 18618-689, SP, Brazil

**Keywords:** protein synthesis, ribosomal biogenesis, gene regulation, CRISPR-Cas9, stress granules, P-bodies, Saccharomyces cerevisiae

## Abstract

**Background/Objectives:** Translation and the formation of membraneless organelles are linked mechanisms to promote cell stress surveillance. LncRNAs responsive to ethanol stress transcr_9136 of the SEY6210 strain and transcr_10027 of the BY4742 strain appear to act on tolerance to ethanol in these strains. Here, we investigate whether the ethanol responsiveness of transcr_9136 and transcr_10027 and their role in ethanol stress are associated with protein biogenesis and membraneless organelle assembly. **Methods:** SEY6210 transcr_9136∆ and BY4742 transcr_10027∆ and their wild-type counterparts were subjected to their maximum ethanol-tolerant stress. The expression of the transcr_9136, transcr_10027, *ILT1, RRP1, 27S, 25S, TIR3*, and *FAA3* genes was accessed by qPCR. The level of DCP1a, PABP, and eIF4E proteins was evaluated by Western blotting. Bioinformatics analyses allowed us to check whether transcr_9136 may regulate the expression of RRP1 and predict the interaction between transcr_10027 and Tel1p. The cell death rate of SEY6210 strains under control and ethanol stress conditions was assessed by flow cytometry. Finally, we evaluated the total protein yield of all strains analyzed. **Results:** The results demonstrated that transcr_9136 of SEY6210 seems to control the expression of *RRP1* and *27S rRNA* and reduce the general translation. Furthermore, transcr_9136 seems to act on cell membrane integrity. Transcr_10027 of BY4742 appears to inhibit processing body formation and induce a general translation level. **Conclusions:** This is the first report on the effect of lncRNAs on yeast protein synthesis and new mechanisms of stress-responsive lncRNAs in yeast, with potential industrial applications such as ethanol production.

## 1. Introduction

Ethanol tolerance is the capacity of cells to withstand prolonged exposure to ethanol. The highest level of ethanol tolerance for a particular strain is the highest concentration at which post-stress growth is still permitted [1,2].

Long noncoding RNAs (lncRNAs) are noncoding transcripts longer than 200 nucleotides in length. At least 75% of the *Saccharomyces cerevisiae* genome encodes RNAs, including more than 500 lncRNAs that control multiple pathways [3,4,5,6,7,8]. Most yeast lncRNAs studied are involved in transposon silencing, telomere replication, osmotic stress, metabolism, cell aging, cell–cell adhesion, cell wall regulation, mating-type regulation, and other processes [6,9,10,11,12]. Some noncoding RNAs in yeast respond to stressors such as ethanol, temperature, and starvation [13]. Moreover, yeast lncRNAs interact with proteins and RNAs in response to stress [8,14].

Ethanol causes ribosome stalling in *Escherichia coli* and most likely *S. cerevisiae* [15]. Stressed yeast cells promote stress resistance by inhibiting the translation of regular mRNAs, favoring the translation of stress response mRNAs [16]. Processing bodies (P-bodies or PB) and stress granules (SG) are membraneless organelles composed of ribonucleoprotein complexes that regulate translation and induce translation restart following stress [17,18]. By interacting with the decapping enzyme 1a of PB (DCP1) and the poly(A) binding protein of SG (PAB1), mRNAs are stored or degraded [19]. SG inhibits translation by sequestering ribosome-free mRNAs, translation-initiation proteins, rRNAs, and other translation-related molecules [16]. PB and SG assembly and protein translation are interconnected mechanisms in cells [16,18,20,21].

LncRNA–RNA or lncRNA–protein complexes can promote the formation of membraneless organelles such as PB and SG. In vitro studies demonstrate the importance of lncRNA in PB and SG [22] in several organisms: lncRNA THOR is responsible for recruiting the IGF2BP1 subunit in PB, while lncRNA ARlnc1 recruits the HuR subunit [23]; the presence of lncRNA NORAD in SG is essential for the recruitment of TIA-1 and TIAR, which are essential for the structure and functioning of SG [24]; glutamine deprivation induces the expression of the lncRNA GIRGL (glutamine insufficiency regulator of glutaminase) inducing SG [25].

Few studies have examined the stress-response mechanisms mediated by lncRNAs in *S. cerevisiae*, despite interest in stressors of *S. cerevisiae*. We previously found hundreds of ethanol-stress-responsive lncRNAs in six yeast strains (BMA64-1A, BY4742, SEY6210, BY4741, X2180-1A). Most of these lncRNAs act on distinct pathways [8], and cis and in trans regulate other genes [26] in a strain-specific manner.

Ethanol stress induced the expression of transcr_9136 and transcr_10027 in SEY6210 and BY4742, respectively [8]. The inactivation of transcr_9136 decreased population rebound after most of the ethanol stress reliefs tested [27], while the inactivation of transcr_10027 increased population rebound [8]. Transcr_9136 induces a regular cell cycle even under stress with ethanol [27], and transcr_10027 interacts with the Tel1 protein related to PB [8]. Therefore, we investigated whether the ethanol responsiveness of transcr_9136 and transcr_10027 and their role in ethanol stress are associated with protein biogenesis and membraneless organelle assembly.

We used ethanol to disrupt the systems of mutants with partially deleted transcr_10027 and transcr_9136 and their wild-type (WT) counterparts. The results demonstrated that transcr_9136 and transcr_10027 present different impacts on translation. Moreover, transcr_9136 may act on membrane integrity, whereas transcr_10027 may inhibit PB formation.

## 2. Material and Methods

### 2.1. Yeast Strains

The *S. cerevisiae* BY4742 (S288C isogenic yeast strain: MATα; his3D1; leu2D0; lys2D0; ura3D0) and SEY6210 (MATα suc2-Δ9 ura3-52 leu2-3112 his3-Δ200 trp1-Δ901 lys2-801) wild-type strains were obtained from Euroscarf. SEY6210 transcr_9136∆ and BY4742 transcr_10027∆ mutants with inactive versions of lncRNA transcr_9136 and transcr_10027, respectively, were previously generated [8,27]. All experiments used late-log phase cells: strains were grown overnight (~12–16 h) in 20 mL of YPD medium (2% peptone, 1% yeast extract, and 2% glucose) at 30 °C and 200 RPM before experiments.

### 2.2. Stress Induction, Gene Expression, Western Blot, and Analysis of the Hi-C Database

Late-log phase cells were diluted in YPD to an OD_600_ of 0.2 for qPCR and 0.3 for Western blot analysis. Cells were transferred in triplicate to 6-well plates and treated for 1 h with 2 mL (for qPCR) or 20 mL (for Western blot) of YPD with different concentrations of ethanol (*v*/*v*) according to strains (20% for SEY6210 and 26% for BY4742): these conditions were previously selected as the highest ethanol level tolerated for these strains [8]. The same protocol, but using a physiological solution instead of ethanol, was used to obtain the untreated samples. Furthermore, the rebound of the BY4742 WT and mutant strains was carried out by striking cells on YPD plates after 1 h of treatment with different ethanol levels.

For qPCR, cells were harvested (5000 RPM for 2 min) in 1.5 mL microtubes, the supernatant was discarded, and the pellets were stored at −80 °C until use. Total RNA was extracted from untreated and treated samples of WTs and mutants using the SV Total RNA Isolation System (Promega, Madison, WI, USA) according to the manufacturer’s instructions after treatment with 100 µL of lyticase (1 U/mL) for 30 min (Sigma-Aldrich, St. Louis, MO, USA) and 2.88 mM β-mercaptoethanol at room temperature. RNA quality was assessed by electrophoresis on a 1.5% agarose gel stained with GelRed (Sigma-Aldrich, St. Louis, MO, USA) and bromophenol blue, and the RNA yield was estimated by a NanoDrop. Then, 1 µg of RNA was used as a template for cDNA synthesis using the High-Capacity cDNA Reverse Transcription Kit (Applied Biosystems, Waltham, MA, USA) following the manufacturer’s instructions. The qPCR was carried out in biological and technical triplicates using the following reaction: 3 µL cDNA (1:10 dilution), 1.0 µM for the forward primer, 1.0 µM reverse primer (Table 1), and 1X GoTaq ^®^ qPCR MasterMix (Madison, WI, USA) for a final volume of 15 µL. The qPCR cycling was 95 °C for 2 min, 45 cycles of 95 °C for 3 s, 60 °C for 30 s, and 72 °C for 30 s, with a melting curve, using a 7500 Real-Time PCR Systems thermocycler (Applied Biosystems). The *TDH2* gene (YJR009C) was used as an endogenous gene [28]. CT data were analyzed using ∆∆Ct methodology [29]. The statistical analyses were performed using one-way ANOVA with Tukey’s multiple comparisons test using the mean of replicates. Since *25S* and *27S* were quantified in the same plate, we subtracted the normalized expression of *25S* by the *27S* to quantify *25S* because its primer also binds to the *27S* (Figure S1).

For Western blotting, proteins were extracted from control and treatment conditions of mutants and WT strains. First, samples after 1h of treatment and control were precipitated (1000 RPM for 1 min), and the supernatant was discarded. A total of 600 µL of LiAc 2.0 M was added and mixed vigorously. Then, 400 µL of NaOH 0.4M was added and mixed vigorously. Samples were incubated in ice for 5 min. Samples were transferred to 2 mL tubes and precipitated with 3000 RPM for 1 min, and the supernatant was discarded and stored at −80 °C until use. Protein yields were estimated using the Bradford method [30]. Then, 30 μg or 70 μg of proteins were applied in 10% polyacrylamide gel electrophoresis; the molecular weight used was the standard Kaleidoscope (Bio-Rad, Hercules, CA, USA). Proteins were then transferred to a Hybond ECL nitrocellulose membrane (Amersham, Little Chalfont, UK) and blocked for 1 h with 5% fat milk diluted in TBS, followed by incubation (16 h at 4 °C) with primary antibodies that bind to the desired target: anti-DCP1a (sc-100706; Santa Cruz, 1:1000), anti-PABP (sc-166027, Santa Cruz, 1:1000), and anti-Elf4e (sc-9976, Santa Cruz, 1:1000), molecular markers for PB, SG, and translation stalling, respectively [31,32,33]. The membranes were washed in TBS for 2 h and incubated with the specific secondary antibody for each primary antibody, followed by washing using TBS. The chemiluminescence reaction was performed using the ECL kit, and the images were captured using Image Quant 350 (GE—Healthcare). Semiquantitative analyses by band densitometry were performed and analyzed using GraphPad Prism 7.00 software. Expression levels were normalized to β-actin 42 kDa, and normalized results were expressed in fold change. The quantification of each replicate was normalized by dividing by the average of the control within each condition and strain. Then, ordinary one-way ANOVA (Tukey test and swapping directions for comparisons) was applied to assess the significance between control and treatment.

To assess the total protein yield under control and treated conditions of both WT and mutant strains, we performed the same protocol for Western blot quantification. However, before treatment, we quantified total cells and diluted to an OD_600_ of 0.2 in 20 mL of medium with ethanol or physiological solution (20% for SEY6210 and 26% for BY4742): these conditions were previously defined as the highest ethanol level tolerated for these strains [8]. Then, we performed the treatment as already mentioned followed by the same procedures for Western blotting. Proteins were quantified using the Bradford method [30]. The total protein yields were compared by ordinary one-way ANOVA with the Tukey test.

The homologous locus of transcr_9136 of SEY6210 was analyzed in the S288C strain to inspect possible chromatin interactions between this lncRNA and surrounding regions using the public Hi-C database [34].

### 2.3. Molecular Docking

The secondary and tertiary structures of transcr_10027 of BY4742 were modeled using the RNAFold web server (http://rna.tbi.univie.ac.at/cgi-bin/RNAWebSuite/RNAfold.cgi accessed on 29 April 2024) and the 3dRNA/DNA web server (http://biophy.hust.edu.cn/new/3dRNA/create accessed on 29 April 2024), respectively, using default parameters.

Blind docking between transcr_10027 and Tel1p (PDB number 6JXC) was performed using the HDOCK [35]. To reduce the number of atoms in the system, a simplified model was constructed containing only the interface region of transcr_10027 with Tel1p (Figure S2B).

### 2.4. Flow Cytometry

For flow cytometry analysis, SEY6210 WT and SEY6210 transcr_9136∆ strains were treated with 20% ethanol as previously described. Then, 1× 10^6^ cells were washed in 1× PBS and centrifuged at 14,000× *g* for 1 min. The pellet was resuspended in 1 mL of 1× binding buffer. A total of 100 μL of mixed solution was transferred to a new tube with 5 μL of propidium iodide. The mixed samples were incubated at 25 °C for 15 min in a dark chamber. Cells were resuspended using 400 μL of 1× binding buffer. The samples were immediately measured using the BD FACSCalibur™ analyzer, and the data were analyzed using the BD CellQuestPro software (BD Biosciences, Franklin Lakes, NJ, USA). The analysis was performed using four biological replicates for each strain. Data were analyzed by the *t*-test (two-tailed test) to compare WT and mutant strains.

## 3. Results

### 3.1. Rationale

We previously determined that BY4742 and SEY6210 support 20% and 26% ethanol levels (volume/volume (*v*/*v*)), respectively [8]. Therefore, we used these percentages of ethanol for the experiments performed here.

LncRNAs may cis-regulate the expression of nearby genes [36,37]. We analyzed by qPCR whether the lncRNAs transcr_9136 and transcr_10027 regulate the expression of adjacent genes in response to ethanol stress.

DCP1a, PABP, and eIF4E were previously used as molecular markers in yeast for the study of PB, SG, and translation, respectively [38,39,40,41]. We investigated the effects of lncRNA transcr_10027 and transcr_9136 on protein synthesis and membraneless organelles by qPCR and Western blotting. Thus, we measured the levels of DCP1a, PABP, eIF4E, 27S pre-rRNA, and 25S rRNA in SEY6210 transcr_9136∆ and BY4742 transcr_10027∆ mutants and their wild-types (WT) stressed by ethanol. Furthermore, we analyzed the expression of transcr_9136 and transcr_10027 in SEY6210 and BY4742, respectively, by qPCR.

Based on qPCR data, we hypothesized that the lncRNA transcr_9136 of the SEY6210 strain could work in cell membrane permeability. We tested this hypothesis with flow cytometry analysis.

### 3.2. Analysis of SEY6210 WT and SEY6210 Transcr_9136∆ Mutant

**Figure 1:**
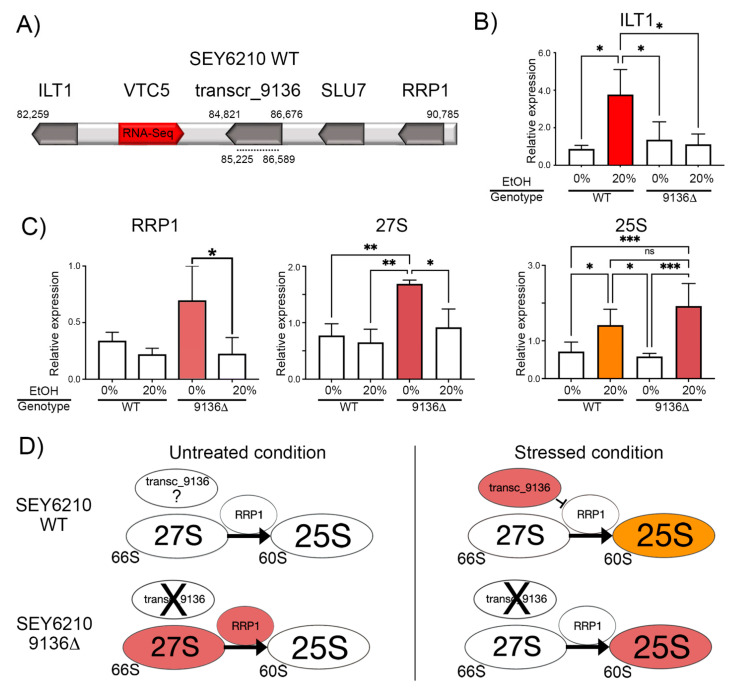
Analysis of gene expression and protein abundance in SEY6210 WT and SEY6210 transcr_9136∆ mutant strains. (**A**)**:** Genomic region of contig gi|696450445|gb|JRIW01000133.1 retrieved from SEY6210 Stanford GFF deposited in the SGD database. The numbers above the bar indicate the start and end positions of the analyzed region and the position of transcr_9136. The dashed line under the bar indicates the deleted region in the mutant. (**B**,**C**)**:** qPCR data. Genes or samples with similar expression levels are represented by the same color bar. (**D**)**:** summarization of the qPCR results of genes involved in ribosomal biogenesis. The colors represent the quantified expression levels denoted in A and C; EtOH: ethanol. *: *p*-value < 0.05; **: *p*-value < 0.01; ***: *p*-value < 0.0005; expression not quantified; X: absence of transcr_9136; The blunted line means inhibition of expression.

Transcr_9136 of SEY6210 is surrounded by *ILT1*, *VTC5*, *SLU7*, and *RRP1* (Figure 1A). We hypothesized that transcr_9136 could cis-regulate the expression of adjacent genes in response to ethanol stress. SEY6210 WT subjected to 20% ethanol (*v*/*v*) increased the expression of transcr_9136 (log2-fold-change of 1.252) and its adjacent gene *VTC5* (log2-fold-change of 1.11) [8]. The qPCR of transcr_9136 comparing the WT under treated and control conditions evidencing a down-regulation of this lncRNA in stressed cells (Figure S3). Using qPCR, we quantified the expression of *ILT1* and *RRP1* in SEY6210 transcr_9136∆ and WT strains disrupted with 20% ethanol (*v*/*v*). *ILT1* increased expression only in stressed WT cells, whereas *RRP1* increased expression in untreated mutant cells (Figure 1B,C).

ILT1 encodes a plasma membrane protein, which confers tolerance to ionic liquids [42]. In flow cytometry assays, propidium iodide is used to verify cell viability by membrane disruption. Therefore, we tested the hypothesis that the observed increased expression of ILT1 in SEY6210 WT cells under stress would improve cell permeability by analyzing the percentage of dead cells (cells with disrupted membrane) of SEY6210 WT and SEY6210 transcr_9136∆ treated with 20% ethanol. The flow cytometry data reported that the percentage of dead cells in the WT strain under stress is lower than that of the treated mutant (*p*-value = 0.016), corroborating our hypothesis (Figure S4).

RRP1 (this gene is in the vicinity of transcr_9136, Figure 1A) catalyzes the conversion of 27S pre-rRNA to 25S rRNA [43,44]; therefore, we chose RRP1 for further investigation due to its crucial role in ribosomal biogenesis. A Hi-C matrix depicts the chromatin organization, revealing the genomic distance among loci [45]. Analysis of previous Hi-C data for yeast [34] revealed that the transcr_9136-like and RRP1 loci in the S288C strain strongly interact throughout the cell cycle (Figure S5), providing preliminary support for our hypothesis that this lncRNA could influence the ribosomal biogenesis.

Then, we hypothesized that transcr_9136 indirectly affects the 27S and 25S without considering a direct lncRNA-rRNA physical interaction. To test this hypothesis, we quantified the expression of 27S and 25S by qPCR. We observed a similar expression profile between 27S and RRP1, with significantly higher expression in the untreated mutant. Surprisingly, the 25S level increased in both treated cells (Figure 1C).

**Figure 2:**
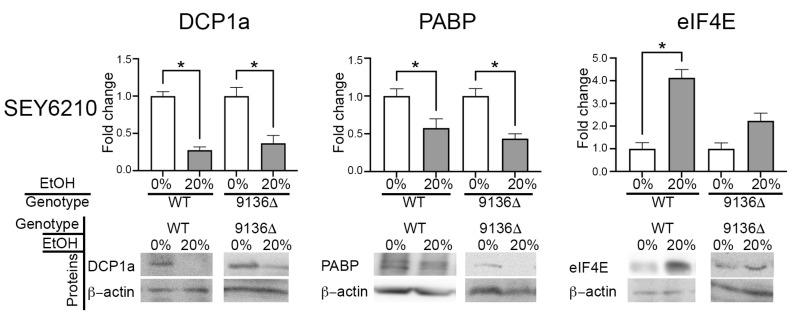
Analysis of selected proteins by Western blotting in SEY6210 WT and transcr_9136∆ mutants. The concentration of each protein was adjusted relative to β-actin and is shown as a fold change. Although double bands in the PABP gel are not an ideal result, they are common for the PABP antibody used here, as described in the datasheet of Wolf et al. 2023 [8]. *: *p*-value < 0.05.

Bioinformatic analysis predicts that transcr_9136 interacts with the stress granule (SG) protein Ecm32p [8]. The primary function of SG is to suppress translation during stress by sequestering rRNAs, translation initiation proteins, and others [16]. Translation repression, P-bodies (PB), and SG are interconnected mechanisms in stressed cells [16,18,20,21]. Therefore, we hypothesized that transcr_9136 may influence PB, SG, and translation repression. Western blot analysis of SEY6210 transcr_9136∆ and WT subjected to 20% ethanol stress revealed a decrease in DCP1a and PABP levels and an increase in eIF4E levels in stressed strains (Figure 2 and Figure S6).

### 3.3. Analysis of BY4742 WT and BY4742 Transcr_10027∆ Mutant

The RNA-Seq data previously published [8] showed that transcr_10027 is up-regulated in wild-type BY4742 under ethanol stress (log2-fold-change of 0.695): indeed, the qPCR of transcr_10027 comparing the WT under treated and control conditions showed an up-regulation of this lncRNA in stressed cells (Figure S3), corroborating our previous transcriptome data [8].

Transcr_10027 of BY4742 interacts with Tel1p (with 96% probability of interacting) [8]. Then, we investigated by molecular docking whether transcr_10027 could indeed bind to Tel1p. The secondary and tertiary structures of transcr_10027 were de novo modeled (Supplementary Data S1; Figure S2A (Supplementary Data S2 is available at https://figshare.com/account/items/28287446/edit; accessed on 27 January 2025)): we chose the lowest tertiary structure (26.5114) score, such as recommended by the 3dRNA/DNA tool (http://biophy.hust.edu.cn/new/3dRNA/create accessed on 29 April 2024). The blind docking between transcr_10027 and Tel1p (Figure S2) showed a docking score of −206.43 and a confidence score of 0.7556: a lower negative score indicates a higher probability of complex formation, and a confidence score >0.7 indicates that the molecules have a high probability of binding [46]. Therefore, our docking between Tel1p and transcr_10027 corroborated a previous prediction concerning their interaction [8]. Interestingly, the Tel1p functional domains were not in the interface with transcr_10027, except by a small portion of the FAT domain.

**Figure 3:**
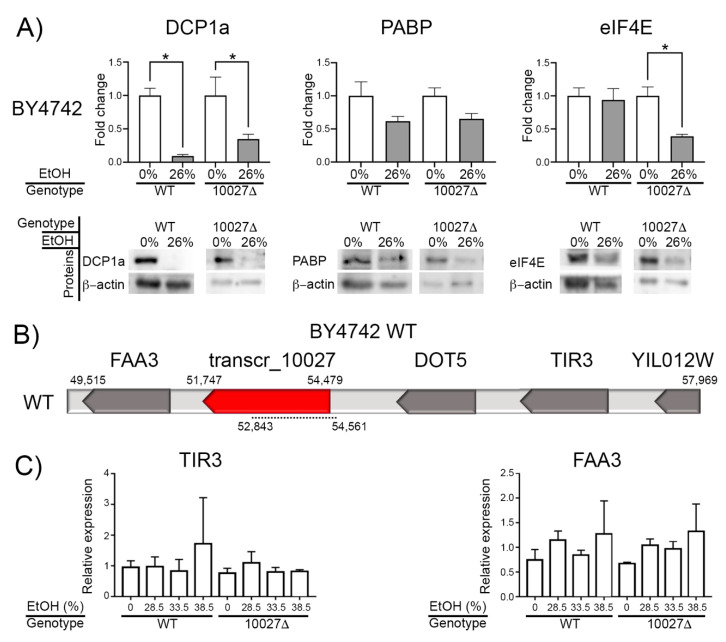
Analysis of gene expression and protein abundance in BY4742 WT and transcr_10027 mutants. (**A**)**:** Western blot analysis measuring DCP1a, PABP, and eIF4E protein levels. The protein yield was adjusted to β-actin and displayed as a fold change. (**B**)**:** Genomic region of contig gi|696446837|gb|JRIR01000161.1 retrieved from BY4742_Stanford GFF deposited in the SGD database. The numbers above the bar indicate the start and end positions of the analyzed region and the position of transcr_10027. The dashed line under the bar indicates the deleted region in the mutant. Red indicates up-regulation. (**C**)**:** qPCR data. EtOH: ethanol. *: *p*-value < 0.05.

Active Tel1p also inhibits the formation of PB [47]. PB interacts with SG in yeast [48]. Therefore, we hypothesized that the ethanol stress response of transcr_10027 indirectly influences the formation of PB and SG through the Tel1p interaction. Then, we investigated the effect of transcr_10027 on PB and SG formation, and translational activity in BY4742 cells perturbed with 26% ethanol (*v*/*v*). Western blot revealed a reduced level of DCP1a and PABP in BY4742 WT and transcr_10027∆ cells stressed by ethanol. The eIF4E yield was reduced only in the treated mutant (Figure 3A and Figure S6).

*TIR1* and *FAA3* surround the lncRNA transcr_10027 of BY4742 (Figure 3B). LncRNA cis-regulates *TIR1* expression in yeast [6]. Tir1p and Tir3p are cell wall mannoproteins [49], while Faa3p acts on long-chain fatty acids [50]. The cell wall protects yeasts from ethanol stress [51]. Long-chain fatty acids appear to improve tolerance to ethanol [52,53]. Then, we analyzed whether the transcr_10027 could be regulating the *TIR1* and *FAA3*. According to our qPCR results, neither the WT nor mutant strains exposed to ethanol exhibited significant expression changes in adjacent transcr_10027 genes *TIR3* and *FAA3* (Figure 3C). Altogether, it seems that transcr_10027 does not regulate the expression of its adjacent genes in either of the tested BY4742 strains.

## 4. Total Protein Yield Quantification

Quantification of eIF4E (Figure 2 and Figure 3A) allowed us to hypothesize an induction and repression of translation in SEY6210 transcr_9136∆ and BY4742 transcr_10027∆, respectively. Therefore, we investigated whether the total protein yield changed in treated and untreated WT and mutant cells. The total protein yield in SEY6210 transcr_9136∆ increased in treated cells compared to untreated WT cells, whereas the protein yield was significantly reduced in BY4742 transcr_10027∆ than in untreated WT (Figure S7), corroborating our hypothesis.

## 5. Discussion

### 5.1. The Action of LncRNAs on Translation

Yeast cells require the induction of ribosomal genes to allow growth, metabolism, and survival during prolonged stress by ethanol [54]. Yeast ribosomes contain several proteins in addition to 18S, 5.8S, 25S, and 5S rRNAs [44]. There is a positive correlation between rRNA and ribosomal yields [55]. rRNA levels are positively correlated with ribosomal assembly: ribosomal biogenesis relies on the expression and maturation of rRNAs, including 18S and 25S [56,57,58,59]. The low expression of *28S* (corresponding to yeast *25S*) in mutant rats appears to compromise ribosomal biogenesis [44,60].

The Rrp1p enzyme catalyzes the conversion of 27S pre-rRNA (a component of the 66S ribosomal subunit) to rRNA 25S (a component of the 60S subunit) [43,44]: the rrp1-1 mutant does not convert 27S pre-rRNA into 5.8S and 25S rRNA, reducing the levels of 60S and 66S [43]. The expression of *RRP1* and *27S* in SEY6210 transcr_9136Δ indicates that this lncRNA seems to control the levels of *RRP1* and *27S* (Figure 1D). However, it remains unclear whether this lncRNA physically influences the specific conversion from 27S to 25S.

Protein synthesis and assembly of PB and SG are interconnected mechanisms in stressed cells. PB and SG contain rRNAs, translation initiation factors, and other translation and mRNA decay proteins. This trapping causes translation stalls, allowing for more efficient translation reactivation [16,17,18,20,21,39,47,61,62,63,64,65,66,67,68]. However, ethanol stress may promote SG assembly without containing eIF4E proteins [16]; the primary function of the eIF4E protein is to recruit ribosomes to initiate mRNA translation, regardless of eIF4E phosphorylation [69,70]. Ethanol stress reduces DCP1a (PB marker) and PABP (SG marker) yields in all strains tested here, suggesting that acute stress stimulates neither PB nor SG formation. Furthermore, drastic changes in eIF4E yield across all strains suggest that ethanol stress significantly affects translation. In fact, we observed an increase in protein yield for the SEY6210 mutant and a reduction for the BY4742 mutant compared to the untreated WTs. Altogether, transcr_9136 seems to reduce translation in SEY6210, whereas transcr_10027 seems to induce translation in BY4742. In this case, the induction of translation in BY4742 by transcr_10027 does not seem to match the eIF4E level, indicating a more intricate regulatory mechanism in this strain.

### 5.2. Transcr_10027 and PB Formation

**Figure 4:**
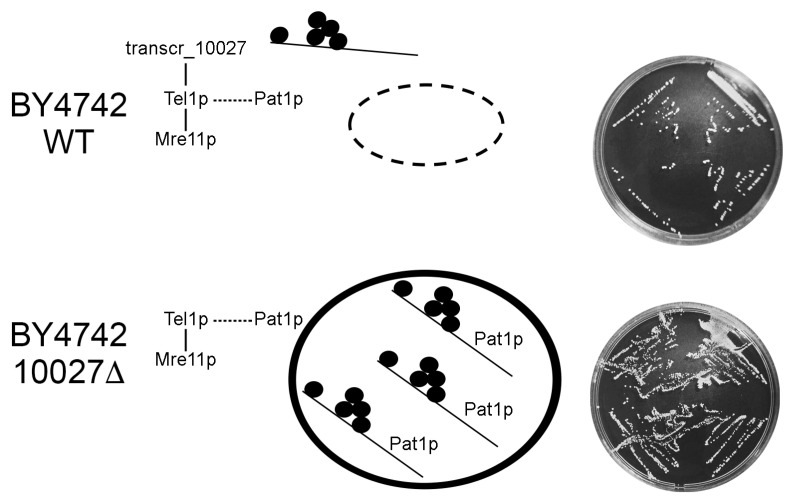
The PB aggregation model in ethanol-stressed BY4742 cells. The small black circles represent mRNA-binding proteins, such as LSMs, DCPs, and Dhh1p. Dashed line circle indicates an absence of PB. Dashed lines linking Tel1p and Pat1p are unknown pathways. Plates on the right show WT and mutant cells grown for 24 h after a 1 h treatment with 24% ethanol.

Tel1p physically interacts with Mre11p [71,72]. Mre11p is required for Tel1p activation and phosphorylation [73]. Mre11p also interacts with Pat1p [74]. The deletion of *TEL1* and its homolog enhanced PB formation. Then, Tel1p represses Pat1p and PB formation, whereas Pat1p promotes it [47,64,75]. The interactions between Tel1p and Pat1p for the formation of PB are still unknown. Trancr_10027 of BY4742 interacts with Tel1p, and ethanol stress increases the expression of this lncRNA. Although the level of the PB marker DCP1a was reduced in BY4742 mutant and WT strains stressed with ethanol, the reduction was greater in WT, suggesting that transcr_10027 seems to inhibit PB formation prompting a lower population rebound in this strain (Figure 4).

### 5.3. Transcr_9136 Seems to Work on Cell Membrane Integrity

ILT1 encodes a plasma membrane protein with an unclear function, although Ilt1p confers tolerance to ionic liquids [42]. Ethanol stress directly modifies yeast lipid saturation [76]. Furthermore, yeast strains subjected to severe ethanol stress, including SEY6210, present a significant change in lipid metabolism [8]. Inactivation of transcr_9136 by CRISPR-Cas9 inhibited the *ILT1* response to ethanol stress, which was up-regulated in WT stressed cells. Moreover, the flow cytometry assay reported a significantly lower cell permeability in SEY6210 WT under stress. Therefore, we suggest that a low level of transcr_9136 in SEY6210 WT cells accentuates *ILT1* expression and might contribute to maintaining cell membrane integrity in ethanol-stressed cells.

## 6. Conclusions

We suggest that transcr_9136 and transcr_10027 present different impacts on translation. Furthermore, transcr_9136 may act on membrane integrity by boosting *ILT1* expression in stressed cells, whereas transcr_10027 may inhibit PB formation. Altogether, a low level of transcr_9136 of SEY6210 appears to aid this strain in coping with ethanol stress, whereas transcr_10027 of BY4742 seems to hinder this process. Our findings provide new information on the physiological mechanism of ethanol tolerance, with implications for the genetic engineering of lncRNAs to improve ethanol tolerance and production. For instance, we wondered whether the induction of transcr_9136 might improve ethanol tolerance.

## Figures and Tables

**Table 1:** Oligonucleotides used in the qPCR analyses. F: forward; R: reverse.

Strain	Gene; Oligo Name	Sequence (5′–3′)	Tm
SEY6210	ILT1; ILT1 F	TTATTGCGGCTGATGTTGGC	60 °C
SEY6210	ILT1; ILT1 R	GCCAAGCACCTAATGAATCG	60 °C
SEY6210	RRP1; RRP1 F	CGTCCTAGACCTCAGCAACG	60 °C
SEY6210	RRP1; RRP1 R	GGTCAACTCATCTGCAGTGCTA	60 °C
SEY6210	27S; 27S F	AGAAGAGAGCGTCTAGGCGA	59 °C
SEY6210	27S; 27S R	CTAAGGCAATCCCGGTTGGT	59 °C
SEY6210	25S; 25S F	GTGAAGCGGCAAAAGCTCAA	59 °C
SEY6210	25S; 25S R	CACACGGGATTCTCACCCTC	59 °C
SEY6210	Transcr_9136 F	CGACAGTAAAGTGAGCAAGG	55 °C
SEY6210	Transcr_9136 R	CAAACGAAGTAAGCGTAGAAAG	54 °C
BY4742	FAA3; FAA3 F	AAACGGCGGTCTCTTTCACT	60 °C
BY4742	FAA3; FAA3 R	GTTGTCCCTTGGGAGTCCAT	60 °C
BY4742	TIR3; TIR3 F	CTCCTCCTCTGCTACCTCCA	60 °C
BY4742	TIR3; TIR3 R	ACCAACACCAGCGGAGAAG	60 °C
BY4742	Transcr_10027 F	CGTCAAGAATCGGAAAGCGT	60 °C
BY4742	Transcr_10027 R	CTTGGTTAGTTTGGGTCGGC	62 °C

## Data Availability

The original contributions presented in the study are included in the article/Supplementary Material, and https://figshare.com/account/items/28287446/edit, further inquiries can be directed to the corresponding author.

## Supplementary Materials

The following supporting information can be downloaded at https://www.mdpi.com/article/10.3390/genes16020170/s1, Figure S1: Details of primers locus to quantify the 25S and 27S rRNAs; Figure S2: Tel1p-transcr_10027 complex with best Docking Score inferred by blind docking in HDOCK; Figure S3: qPCR data of lncRNAs here analyzed expressed in wild-type strains; Figure S4: Flow cytometry assay using propidium iodide; Figure S5: HI-C data of S288C throughout the cell cycle; Figure S6: Uncropped Western blot to quantify the Dcp1a, PABP and eIF4E proteins of SEY6210 and BY4742 wild-types and SEY6210 transcr_9136△and BY4742 transcr_10027△mutants; Figure S7: Total protein yield quantified in control and treated samples; Data S1: the secondary structure of transcr_10027. Data S2: https://figshare.com/account/items/28287446/edit (accessed on 29 April 2024).
