# Supplementary material for: Long Noncoding RNAs Responding to Ethanol Stress in Yeast Seem Associated with Protein Synthesis and Membrane Integrity
Source: Genes (Basel). 2025 Jan 28;16(2):170. doi: 10.3390/genes16020170 (PMC11854924; doi:10.3390/genes16020170)
Supplement: Supplementary file 1 [file genes-16-00170-s001.zip › genes-3332792-supplementary.pdf]

Supplementary Material

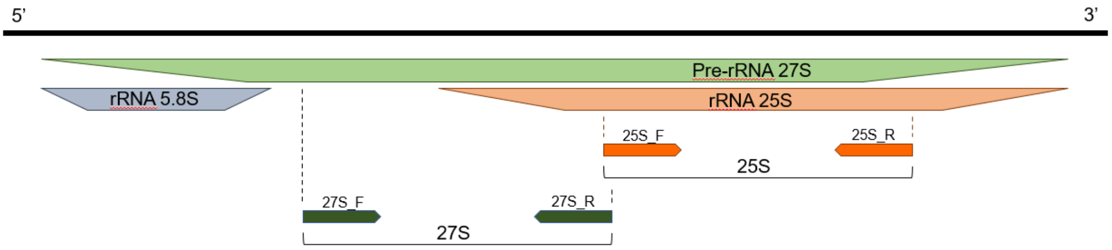

Supplementary Figure S1. Details of primers locus to quantify the 25S and 27S rRNAs.

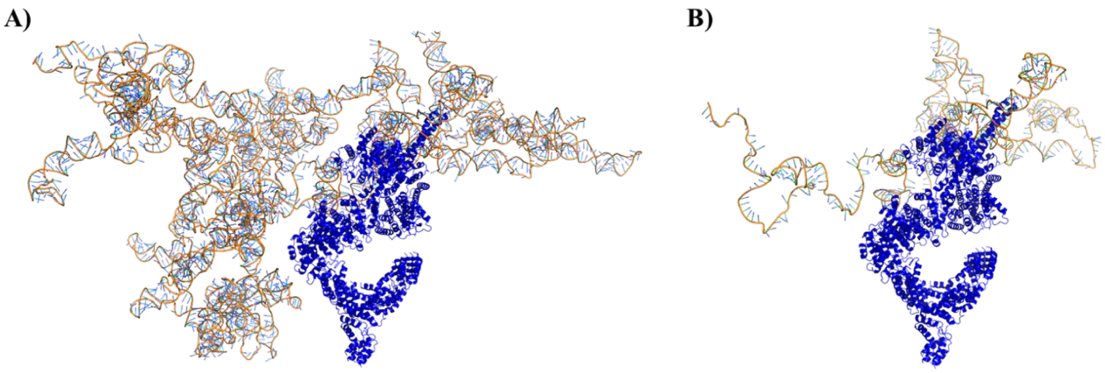

Supplementary Figure S2: Tel1p-transcr_10027 complex with best Docking Score inferred by blind docking in HDOCK. **A:** model with the complete lncRNA; **B:** with the trimmed lncRNA. We trimmed the lncRNA for further analysis due to computational limitations.

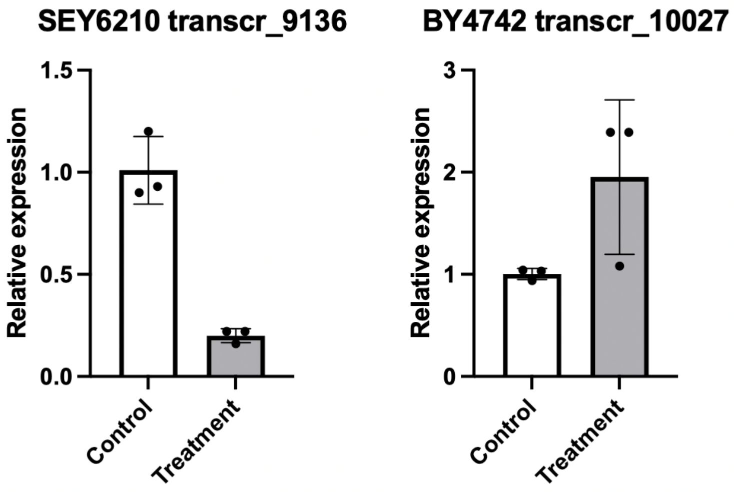

Supplementary Figure S3. qPCR data of lncRNAs here analyzed expressed in wild-type strains.

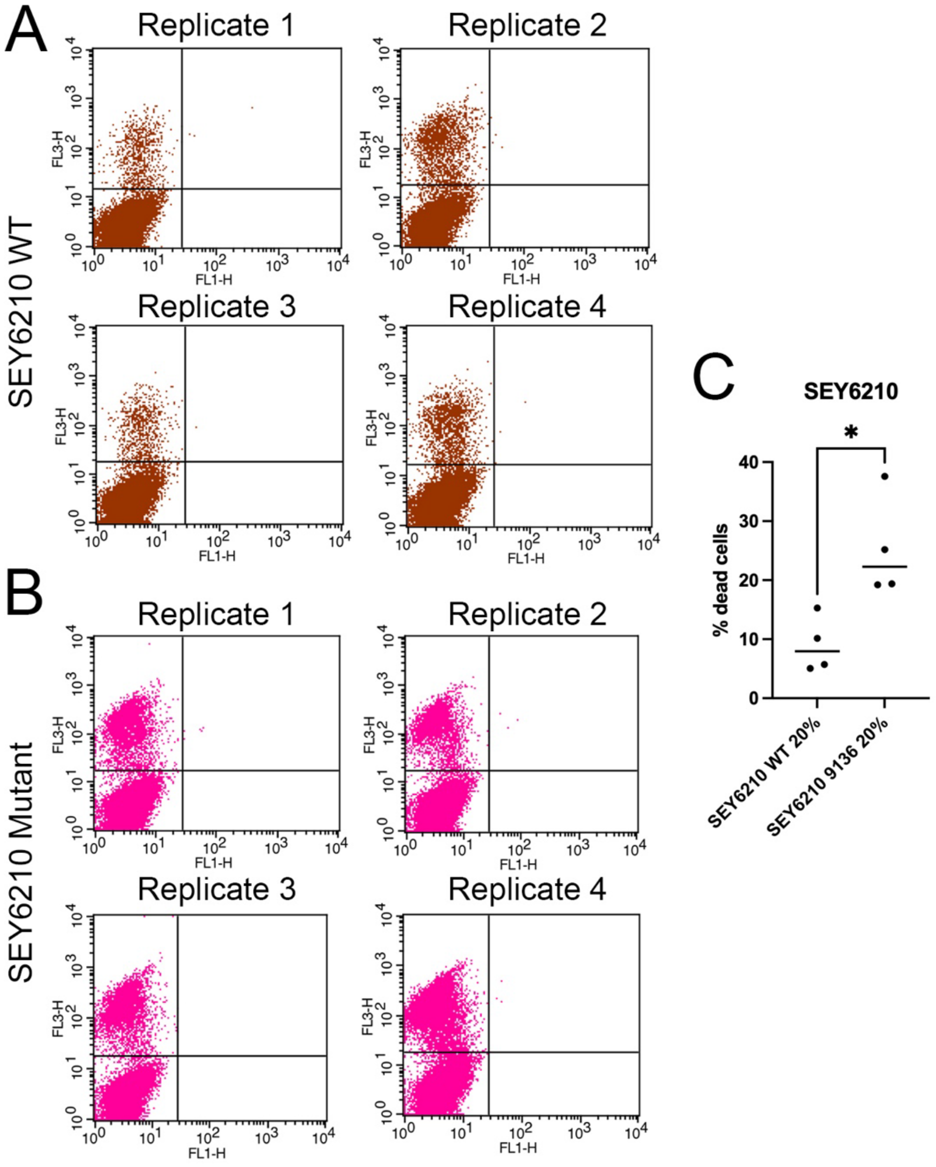

Supplementary Figure S4. Flow cytometry assay using propidium iodide. **A:** data of SEY6210 WT treated with 20% of ethanol; **B:** data of SEY6210 transcr_9136Δ treated with 20% of ethanol; In **A** and **B**, the lower left and upper left quadrants indicate the percentage of viable and dead cells, respectively; **C:** plot of percentage of dead cells in the WT and SEY6210 transcr_9136Δ mutant. *, p-value <0.05.

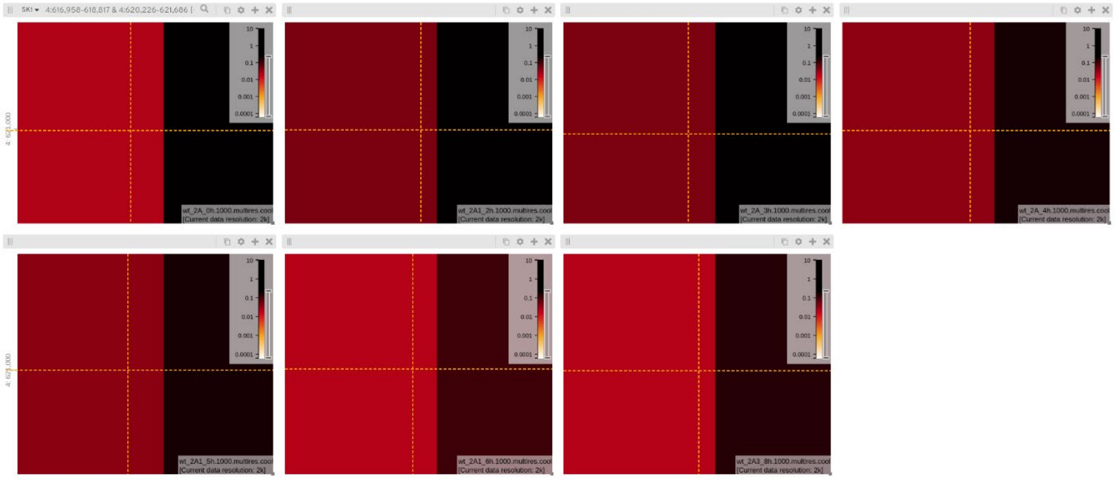

Supplementary Figure S5. HI-C data of S288C throughout the cell cycle. It is shown that locus of transcr_9136-like region and RRP1 locus in S288C. The red color gradient represents the difference between chromatin anchoring between evaluated loci. Each box represents one time-point.

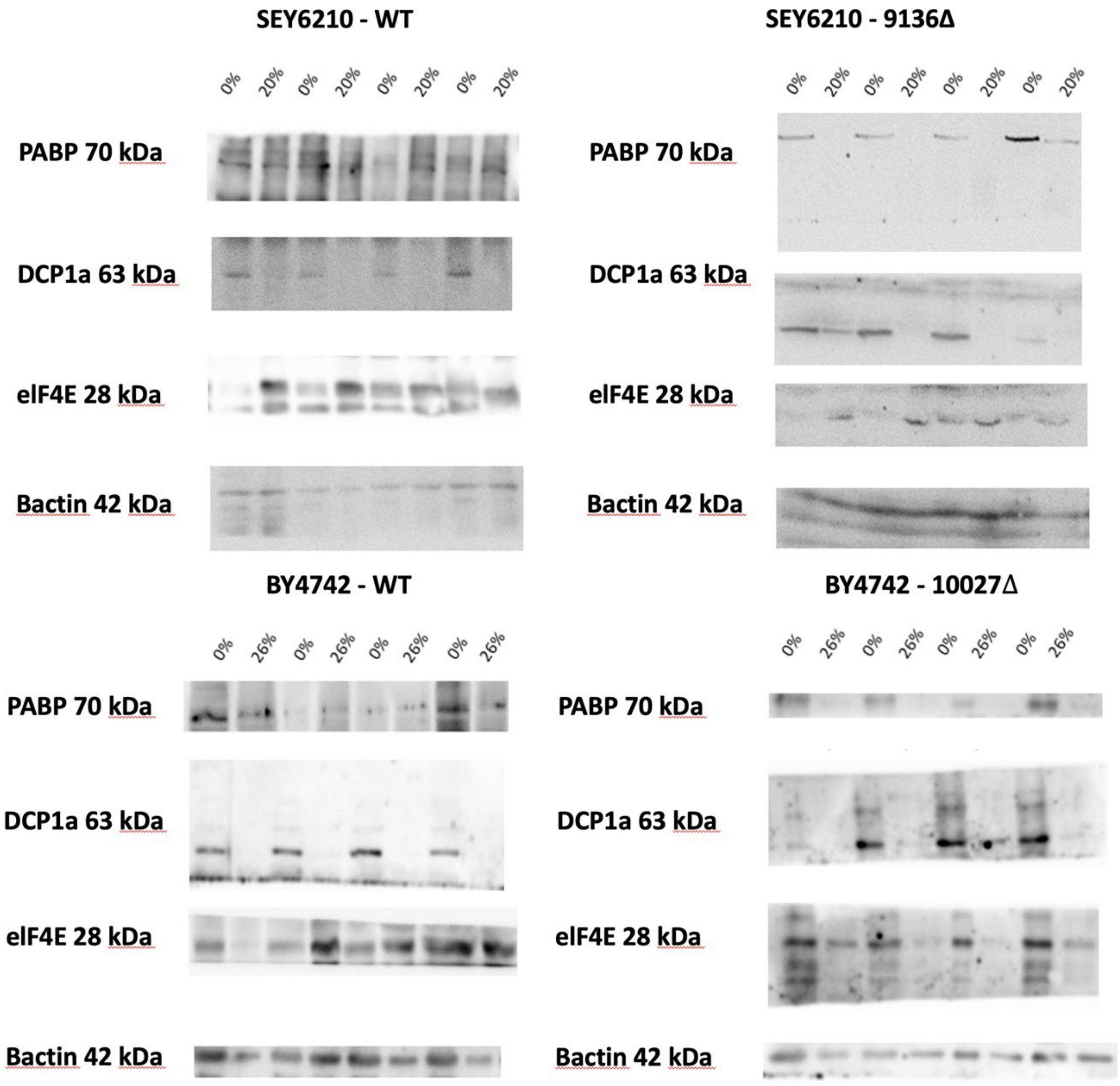

Supplementary Figure S6: Uncropped Western blot to quantify the Dcp1a, PABP and eIF4E proteins of SEY6210 and BY4742 wild-types and SEY6210 transcr_9136△ and BY4742 transcr_10027△ mutants. The number above blot are the percentage of ethanol used in the experiments. Beta-actin is presented as Bactin 42 kDa.

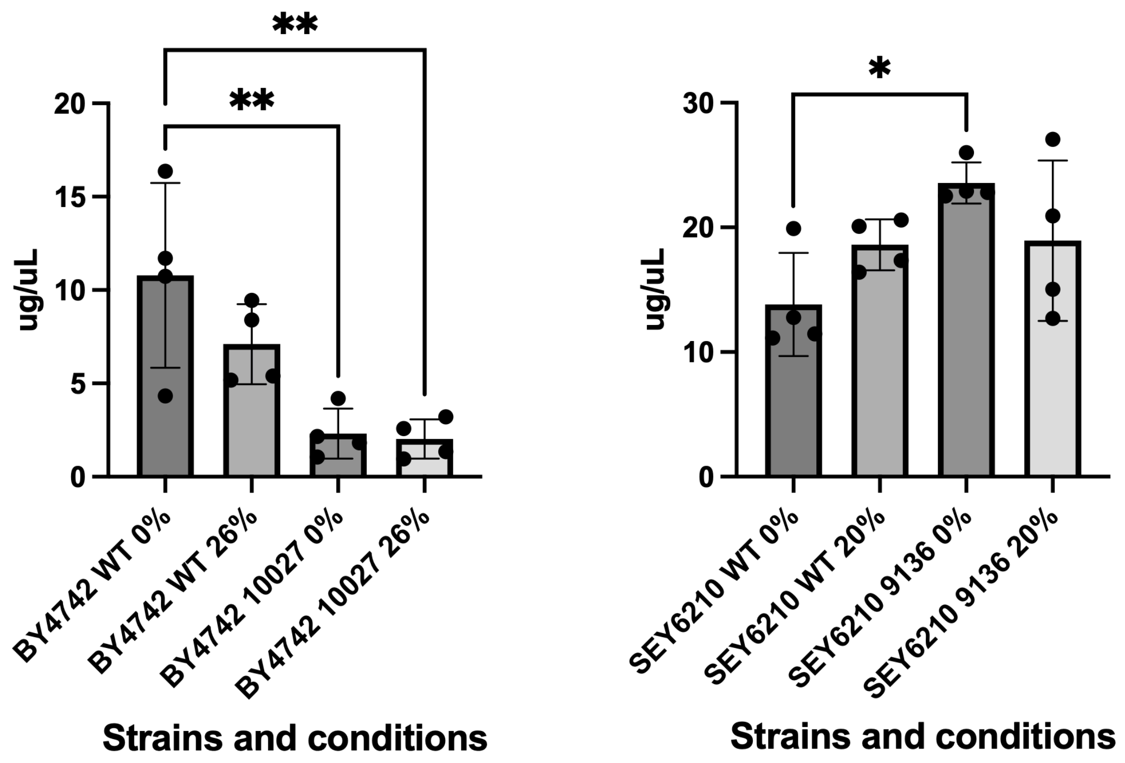

Supplementary Figure S7: Total protein yield quantified in control and treated samples. Asterisks indicate significant differences (p-value <0.05).

Supplementary Data S1: the secondary structure of transcr_10027. The beginning of this file is the sequencing in fasta format, while the brackets, and points in the bottom are codes for the secondary structure.

```
>transcr_10027 of BY4742
AUAACUAGCAGUUUAAAACGUAUCAUUUGUUGAAAUAAGUACCAACUAUCAUCCGUC
GACUAGUACUCACACAACUAUUGAGAAAUUCGUAAGUUUUGUAAAACUGUUGGAUA
CCGUUGCUGUUGAAGGCUAUAGUAAUAGGCAUACAAAAUUAGCGGAAGUUUACCUU
GGAAAUAUAUCCACAAAAGGGAAACGAUAUUUCCUCAUAAUAAUAUUGUAAUUUCUU
UUUCUUUUUAUAUGUUUCAUUAUCCUAUACAUUACCAAUACUUGCAUUUGAGCAUU
CAUUAAAUCUGAUGGGUACAUCUCAAUCUUUCUGUCAUCUUUUUAUAGCAUAUAUG
GUAACAUGCUAGUAGUAUGAAUACUAGUCGAUAUACGUAAAUAAAUUUUCAUUUUAA
AAUGGAUAUGCCUAAUCUGAUAGAUUCUAUUAGCUGAUUUUUCUACUACUUUUCAU
GUUCGUUUUUCAAUAAUUUGCAAGUUAUAUAUCUUUGCCUCCGCUAUACCGUGAUG
AAGGUUAAUGCGUAAGUUGCAGGCGAGGCAUGAUAUGGACCACAACCCAGUUCGAA
CAUAAAGAACUGAAAAACCAUGAGCUGUUUGAUUAAUUCAAAACAGCUGCUCACAAU
UUGGCCCAAGUUCAAACCUUUGUCUUUGAAGCAUGGAACGUGCCGUUAUUUUCAUG
CUAGUGUAAUUUUUGCCUGUUUCAUCCCUCCCAUCAAAAAAUUCAUCACGAAGUGC
UAAAGGAAAACAAAAAAAAUAUCAAAGAGAGGAGACGGCUUAAAAGCCCUUAUUCAA
UUGAAGAAAUCCCCUGACCUCUGCAAAAAAUCUAUCGUUUAUUUUAUUUCUUUGUC
```

UUACGUAACAAAUUUAUUUUCUGGGAUGAUGUCAAUUCUAUUUGGCACGCGAGUCA
UCGUCUAUAUAAGGAGGUAACGAGGUCCUUGUAUAUUUGAUGAUCCUCACUAUCAA
CACGUCGAGGGACGGUUGGUGACGAACUCAACUUAUGUUAUAGUUUUGGACCACU
CUUUUCCUUGAAAAAUUACAGGCAUUAUACGCAGUCCUGGGGCAGGAGCGCUUCGA
UCAUUAGCAUAACAGGAUCACGGUUAUCACCUGUUUUGUUAUAAUUCAUGGCCUUU
AGGUCAUGACGUAAAAUAAUCGGAGUGAAAAAAUGAAAUCAAAGGCUAUCAUUUUUA
GGGGAACUAAAGCACAAGGAUUUGAAGGCGUAGUGAAAUAAUGAAUAAUAGAUAAU
CUAUGGACCGAACACAGACGUAUUUAAAGACAAUACAAGGCCAUUAAUUCCCUUCG
GAGGGCUCCCGAUGGGAGGAUUCUUAGCAUGCUCUUUCUAGCCAACAGUAUCACU
UUCAUCAGAAAACGGAAGCCUCCAAAAAGCUACGCUAAACGCUUUUUUGUUUUGGA
UUAUCGCAGAAAAUUGGUGACAUUCUUGAUUAAGAAUAAACGGUGACUAGCAAGAA
AAGGACAUGUCAGGUACCAGUAAAGUCCGAUUUAAGAUCAAAUCAGCGCCUUUAAA
GAAUCUUGAGAUCUUAGUAAUCCCGAUGGAAGGAAAGAGGUAAACAAACGAAUAAC
UUAACCCUUAAGAUGAUUCUAUACAUAGCAUGAUCUAUCCAAGGAACGACCGACGC
CCUUAAAAGGGUUCUGCUCAGAUUUCGUUAAAUACCCGGACCAUCGUAGAUGAUUG
UUUUUACUCUCCCCUAACGAUGUACUAUGCCAUAAUAAGCAUUUUUUUUUUCUUUC
UUCACUGCAGCUCUGCUUUGAAAACACUACAUCUAAGGCGCUGACGCCAACGCCCA
GACUAAAUCAUCAUAUAAUAUCAAACGCCCAACAAAAUAUUAGAAAUAGCUCAACAA
UAGAGCGCCAAAAGUUUACCGGAACGAACCAUCAUUGUUUACAGGCAAGCACCCAU
UUGGAAGGGUACGUAAACUAAAUAAAUAAGACAAGAAAUUUUUUUUAUAUCUGGAAU
UUCCUUUGCAUUUUAGUGAUCCCACCAUUACAGAUAACUAAACCAGGAUAAAAAACA
AAAAUAAUGACAUUAAUGAUACCUUAACUAACGUAUAGCAAGCGAAGCAACCACCCA
UCAGAUUUUUGUCCCCGUUUGGAAACUGGAAUGCUAUGACCACUUUUAUCACUACA
GCUAACCACUUGUCGGAAUGAAGAUACGAAAGAGAAAUCUUUAUUGGACCACUUUU
UCUUUCGCUAGAGGUCCUACCUAGCGUAUUGCACCCAAGUGAUUAUUGCGACCUCU
GAGUGUCAGAAACUUACGAAGUAAAAUUCAUAGUUGUGCCUUUCGUCAAGAAUCGG
AAAGCGUAAAAAUAGGCUACGGUCUUUGUCAAGGCGAAAUUUUGGAGAUUUCAGAC
GCAUGAUACCAGCAGUUUUUGGUAAUAUACUUUUUCAGGUAAAAGCUUUCUACGGC
CACUGAAAGUAAUGCAGAAGCUUAAGGCUACAGAUGAAAUGGCCGACCCAAACUAA
CCAAGCACAACAUCAAUUACAGAACCGACAAUCGAAAAUCCUAAAAAAAAAGAACAUC
GAGUGAACUUUUCGUUAUCUCAAAGCUUUAAAGUUAAAAGUGGCAGAAGCCUUGGC
UAUUCUGUUUCUCUCUUAGAUUCUCGCACGACAGCAUCGAGCGAAGUUGCAAAACC
AUAUAUUUCAGUUCUCUAAACGAUCUUUCCCCGAACUCCAAUCGAAAUAGACUUAAC
CCGCAACAUAACUGACAUUCUUCCUCUUUUUUUU
....((((((((((((((...(((...((((.....(((((....................)))))....))))...)))...........))))))...)))))))))...((((((((((........)
))))))).))............((((((((((..((((((((((((....((((((((((((((((..........))))))))..))))))))))......)))))))))).................
......(((......)))......((((((((((((........(((((..((((((..((((.((((.....(((((.((((((...))))))..)))))(((...((((((.........((((((.
...))))))..........(((.(((((((((((((.......((((.(((((((((((.(((((((((((.((((((....))))))(((((..((((((.(((..(((........)))...((((..
...)))))))))))))).)))))((.........))(((((..........))))))))))))....((((((((((.....)).))))))))..........(((((...(((((...(((..(((((((((
((((((((.(((....))))))))))))).(((((.....(((((((.(((((((..........(((((....((((((.((.((((((((((..............(((((((.((.((((....)))
)....(((....)))....)).)))...))))...............)))........))))))))))))).)))....)))))........)))))))(((((((......))))))...(((((((((..((((
((((((..........)))))))))))..))))))..)))............((((((((.((.((((...(((((.(((....)))..)))))...))))..)).))))))))......))))))))...)
))))..(((((...))))).(((((((((...(((((.((((((..............)))))).)))))....((((((((....))))))))..........))))))))).............))))))))
...........))).))))))...)).))))))).)))))).))))))))))........)))))))..))))))))))...........))))))).....)))......((((...........)))).(((((..((
(.((((((((((((.((.......(((.......)))....)).))).)))))))))........)))..))))))......)))).((.....))((((((((((((((.(((............(((((..((((((
....))))))....(((.(((((.....((((((.((.((((((((........(((..(((((........((((((((((..((((.((....))))))).........((((((((((((((((((...
.(((((((..........((((.((((.((.(((....))).)).))))....))))...((.((..(((((....)))))..)).))....)))))))...)))).((..((((((((..((..((....))
..))..)).)))))))..))((((..........))))....))))))))))((((..(((....)))..))))........)))).))))))))))).((....))...................((((((......
.........))))))......((((........))))........((((.((.(((((((........)))))))...)).))))((((.........))))............)))))..)))..((((....)))).
))))))).)).)))))).....))))).))).)))))...........))).)))))))))))))..))))..)))))))..)).)))...........(((........)))........)))))))))))

)).......(((((((((((((((((.((((.(((((((((....((.((((......(((((((((((((((((...........)))))))))).........(((((..(((((((((((.......
(((.....(((....))).....)))))))))).))))..))))).....(((......)))...(((((((.(((((........)))))(((.((....)).)))..((((..(((((..(((....(
(((((...)))))))))).))))).....((((((((......(((((((...(((...((....))...)))...)))))))...))))))))...(((((.........)))))))))...........
)))))))............)))))))..........................(((..(((.....)))..)))..)))).))....)))))))))((((((((....)))..)))).................(
(((.(((.....))).)))).((((....)))))))))))))))))).))))))))...................)))..)))))))...))))............................

Supplementary Data S2: https://figshare.com/account/items/28287446/edit
